# Mixed Comparative Evaluation of a Training Program Dedicated to Cystic Fibrosis Reference Centers: Protocol for the Pilot Implementation of Shared Decision-Making in the Treatment of Diabetes in Adult Patients With Cystic Fibrosis

**DOI:** 10.2196/62931

**Published:** 2025-01-28

**Authors:** Nora Moumjid, Constance Gotte, Sophie Hommey, Stéphanie Poupon Bourdy, Julie Haesebaert, Isabelle Durieu, Quitterie Reynaud

**Affiliations:** 1 Département Prévention Cancer Environnement Centre Léon Bérard P2S UR 4129 Université Claude Bernard Lyon 1 Villeurbanne France; 2 Pôle Santé Publique Service Recherche et Epidémiologie Cliniques Hospices Civils de Lyon Lyon France; 3 Research on Healthcare Performance U1290 Inserm Lyon 1 University Hospices Civils de Lyon, Pôle Santé Publique Service Recherche et Epidémiologie Cliniques Lyon France; 4 Cystic Fibrosis Center, Department of Internal Medicine, Hospices Civils de Lyon Research on Healthcare Performance U1290 Inserm Lyon 1 University Lyon France

**Keywords:** shared decision-making, implementation, training, decision aid, cystic fibrosis

## Abstract

**Background:**

Diabetes affects half of the patients with cystic fibrosis who are aged 30 years and older. Diabetes progresses asymptomatically over a long period of time. Two treatment options are possible: start insulin as soon as cystic fibrosis diagnosis is made with the additional constraints of cystic fibrosis or wait while monitoring the patient’s clinical condition and start insulin when diabetes symptoms develop and therefore later. This situation is particularly well suited to shared decision-making (SDM) between the physician (health care team) and patient/relatives.

**Objective:**

The aim of this study was to perform qualitative and quantitative analyses for evaluating the outcomes and experience of SDM implementation between the physician/health care team trained for SDM and patients/their relatives for cystic fibrosis–related diabetes.

**Methods:**

A quasi-experimental with a comparison study will be developed. Three cystic fibrosis reference centers (CFRCs) will be trained in SDM by using a web-based training, including a validated decision aid and coaching for physicians and the medical team. Two control CFRCs will maintain their usual practices. A qualitative analysis through observation of consultations, individual semistructured interviews with patients, and focus groups in CFRCs will be conducted based on a thematic content analysis. Questionnaires related to decision-making and experience of decision-making with and without SDM implementation will be administered to patients and physicians.

**Results:**

Forty patients will be included (8 patients in each center), that is, 60 consultation observations (2 consultations per patient in the intervention groups given the modalities of the SDM process) will be conducted in 2025. Eight focus groups will be conducted in the 5 centers (2 groups in each intervention CFRC and 1 group in each control CFRC). This qualitative corpus plus responses to the patient and physician questionnaires will make it possible to know whether the practice of SDM in CFRCs is increased by an implementation strategy and to analyze the experience of patients and their relatives regarding decision-making modalities. Analysis of the outcomes and experience of the implementation of SDM are of importance to identify the facilitators and barriers to SDM from patients’ and CFRCs’ point of views.

**Conclusions:**

Our study will give us keys to adapt, improve, and disseminate SDM more widely in the context of cystic fibrosis therapy. SDM could thus be used in routine clinical practice in CFRCs at the national level.

**Trial Registration:**

ClinicalTrials.gov NCT04891159; https://clinicaltrials.gov/study/NCT04891159?id=NCT04891159

**International Registered Report Identifier (IRRID):**

PRR1-10.2196/62931

## Introduction

### Background

Many patients want to play an active role in their own care [1], and many health care professionals want the same for their patients [2]. Shared decision-making (SDM) in the physician-patient encounter is one way of meeting this wish. In SDM, “the information exchange is 2 ways […]. The defining characteristic of deliberation […] is its interactional nature (ie, between the physician and the patient or potential others), and both parties work toward reaching an agreement and both parties have an investment in the ultimate decision made” [3].

SDM often relies on information and decision support tools (decision aids), which provide written support for exchanges. Decision aids are increasingly digitalized and adapted to patients’ health literacy levels [4]. They present management options with their benefits and risks, informing patients and enabling them to participate in the decision-making process if they wish so. They can be used both during and outside the consultation. For several years now, national and international health care policies have been encouraging the implementation of SDM for reasons ranging from ethical imperatives [5] to the reduction of unjustified variations in clinical practice [6]. However, despite the legal framework encouraging SDM in several countries (France [7], Germany [8], United Kingdom [9], United States [10]), the implementation of SDM in clinical practice is not yet widespread [11]. SDM research has focused on physician- or patient-mediated interventions to promote SDM [12]. Légaré et al [13] in their Cochrane review showed that a combination of both physician- and patient-mediated interventions is more likely to be successful. With regard to physician-mediated interventions, SDM training can teach health care professionals to involve patients in the decision-making process. A number of studies have shown the positive effects of training programs, such as improving physicians’ SDM skills and increasing patient participation and satisfaction [14,15]. Joseph-Williams et al [16] in their systematic review also showed that patients want to be informed so that they can play an active role in decision-making. Patient-initiated interventions can also enhance the patient’s ability to participate in the decision-making process. Patients who have learned to use tools that encourage them to ask questions to health care professionals participate more actively [17,18]. Finally, decision aids are a means of increasing SDM. The Cochrane systematic review by Stacey et al [4] based on 105 randomized controlled trials showed the beneficial effects of decision aids in improving doctor-patient communication (without increasing patient anxiety), improving patient knowledge, respecting patient rights, increasing physician and patient satisfaction, improving the quality of care, as well as reducing the decision-making conflict.

In 2017, we developed a decision aid based on the Ottawa Personal Decision Aid Guide [19] and the International Patient Decision Aid Standards Collaboration criteria [20] on the theme of cystic fibrosis–related diabetes treatment. Psychometric tests performed at the cystic fibrosis reference center (CFRC) in Lyon showed that the tool was valid and reliable [21]. In recent years, initiatives have been developing to implement SDM in routine clinical practice. Elwyn et al [22] in their systematic review of studies of decision support interventions concluded that the majority of studies do not base their design on an implementation theory or model. Although their review focused on the routine implementation of decision support interventions to promote SDM, there are other strategies for implementing SDM. For example, a large-scale, multicomponent SDM implementation program involving training of health care professionals in SDM and decision aids has been performed within the National Health Service in the United Kingdom (Making Good Decisions in Collaboration [MAGIC] Program). It showed that successful implementation of SDM in routine clinical practice (primary care, urology, obstetrics, oncology) was based on considering stakeholder attitudes, involving all stakeholders at an early stage and analyzing barriers and facilitators [23]. Such an approach is in line with the recommendations for implementation research developed by Grol and Grimshaw [24].

According to us, SDM seems to be particularly relevant in cystic fibrosis, where there are complex treatment options with variable short, medium, and long-term side effects, and where the disease and its treatments have a high impact on the patient’s quality of life. This genetic disease affects almost 7000 people in France [25] and requires lifelong multidisciplinary care. From the moment of diagnosis, patients are regularly monitored in CFRCs. As a result, the doctor, the multidisciplinary team, and the patient/relatives have often known each other for a long time, forging a strong relationship based on mutual understanding and confidence.

A patient’s quality of life is severely impaired by pulmonary exacerbations and regular digestive disorders. The patient’s care load is considerable, combining daily respiratory physiotherapy, daily aerosol therapy, and multi-daily drug treatments. The social constraints (follow-up appointments every 3 months, daily medication intake, diet) associated with the disease and its treatment are considerable [26]. In addition, complications arise during the course of the disease such as diabetes. After the age of 30 years, half of all patients develop diabetes [27,28]. Diabetes adds significant morbidity [28,29]. Cystic fibrosis–related diabetes is quite specific, differing from type 1 diabetes and type 2 diabetes. It has the particularity of being asymptomatic for a long time, with normal fasting blood glucose levels. For this reason, international recommendations suggest annual screening for diabetes from the age of 10 years, with an oral glucose tolerance test [30]. If diabetes is confirmed by the results of the oral glucose tolerance test, the question arises of how to treat it with insulin. If the patient’s clinical condition is stable and the fasting blood glucose level is normal, there are 2 possible treatment options: start insulin as soon as the diagnosis is made or defer initiation of insulin therapy and reserve it for cases where the patient is experiencing impaired respiratory function, increased frequency of exacerbations, or weight loss. Each of these options is complemented by the appropriate dietary and exercise measures generally recommended for all patients with cystic fibrosis. To our knowledge, very little work has been done on SDM in cystic fibrosis [31] and none on SDM for the treatment of cystic fibrosis–related diabetes.

### Research Assumption

We hypothesize that the practice of SDM by CFRC health care teams for the decision to treat cystic fibrosis–related diabetes will be favored by the implementation of an SDM intervention based on e-learning training integrating an information and decision support tool and coaching for physicians and the medical team.

### Aims

#### Primary Objective

The primary objective is to evaluate the pilot implementation of an SDM intervention, that is, the adoption of SDM between the health care professional and the patient with cystic fibrosis–related diabetes among patients managed in centers receiving the intervention compared with patients managed according to the usual decision-making practice in control centers. SDM is a decision-making process in which the health care professional (plus medical team) and the patient (plus his/her relatives) exchange information on treatment options and then reach a common agreement on the decision to take. It is based on an information and decision support tool used during the consultation. The SDM intervention evaluated comprises 5 components.

1. Web-based SDM training (e-learning) for the entire CFRC medical team
2. Individual coaching for physicians and medical team
3. SDM implementation: (1) consultation 1, including patient activation and delivery of the information and decision support tool to the patient; (2) consultation 2 on discussion and decision-making; and (3) between the 2 consultations, the patient has a cooling-off period of 8-15 days, wherein the physician discusses the content of the consultation with the CFRC team
4. Link with institutional patient engagement initiatives
5. Integration of SDM into CFRC multidisciplinary concertation meetings

#### Secondary Objectives

The secondary objectives were to evaluate the effects of SDM on patients’ level of knowledge of management options with benefits and risks, anxiety, and decisional conflict compared with patients managed in centers without intervention and to evaluate the effects of SDM on the physician-patient relationship in terms of information and decision-making.

#### In Intervention Centers

The objectives were as follows.

1. To evaluate the implementation of the SDM approach.
2. To evaluate the experience of the implementation of SDM regarding the treatment of diabetes in patients with cystic fibrosis. For patients: observations of SDM consultations, individual semistructured interviews based on an interview guide containing key items relating to information and participation in decision-making (barriers, helps) based on our previous work in the field, and questionnaires (9-item Shared Decision-Making Questionnaire [SDM-Q-9], CollaboRATE, knowledge, State-Trait Anxiety Inventory, SURE [Sure of myself, Understand information, Risk-benefit ratio, Encouragement] questionnaire). For health care professionals: focus groups with CFRC teams held at the start of the intervention and afterwards in each CFRC in the intervention group.
3. To identify the individual and organizational factors that influence the implementation of SDM in the treatment of diabetes in patients with cystic fibrosis by health care professionals: focus groups with CFRC teams, semistructured interviews with patients, and exchanges with institutional decision makers such as quality services.

#### In Control Centers

The objective is to describe the process and experience of making decisions about diabetes treatment. For patients with cystic fibrosis, in usual practice, the objective is to conduct observations of consultations dedicated to the discussion of insulin therapy, individual semistructured interviews, and questionnaires. For CFRC health care professionals, the objective is to conduct focus groups with teams from each CFRC (focus groups held once in each CFRC during the course of the study).

## Methods

### Ethics Approval

This study has been approved by the Scientific and Ethical Committee of the Hospices Civils de Lyon University Hospital on the data study (outside the Jardé law; MR004-ID 21_356).

### Design and Setting of the Study

Based on our previous work on SDM in cystic fibrosis [21] and the Consolidated Framework for Implementation Research of Damschroder et al [32], this quasi-experimental here-elsewhere study compares the populations of 3 centers receiving the intervention with those of 2 centers not receiving it (controls). The 5 centers will be studied simultaneously to reduce the risk of contamination of the control group. The evaluation will follow a mixed methods approach, combining qualitative methods (consultations observations, individual semistructured interviews with patients, and focus groups with health care professionals) with quantitative methods (self-administered questionnaires). A convergent mixed methods approach will be used, with concomitant data collection, separate analysis, and subsequent linking of results. This study conforms to the StaRi (Standards for Reporting Implementation Studies) checklist (Multimedia Appendix 1).

### Characteristics of the Participants

#### Patient Inclusion Criteria

The patient inclusion criteria were as follows: (1) older than 18 years, with cystic fibrosis, followed up in one of the 5 CFRCs; (2) be able to understand French; (3) have an oral glucose tolerance test at the stage of diabetes or with a blood glucose Holter considered by the clinician to be pathological, justifying possible insulin initiation; (4) have normal fasting blood sugar levels; (5) stable clinical pulmonary and nutritional status, enabling the 2 treatment options to be considered; and (6) having received the information and not exercised their right to object.

#### Patient Exclusion Criteria

Patients who had undergone a transplant and those receiving insulin therapy were excluded.

#### Inclusion Criteria for Health Care Professionals

Medical and paramedical professionals working in the adult CFRC (physicians, nurses, dietitians, psychologists, physiotherapists, etc) who have not exercised their right to object were included.

### Description of Intervention and Comparisons

#### SDM Implementation Program

This program was divided into several parts and as already mentioned, theoretically based on the Consolidated Framework for Implementation Research [21] and on existing literature on the implementation of SDM [13], structured into 5 components (A to E) focusing on health care professionals and patients.

##### Component A: Health Care Professionals’ SDM Training in the 3 CFRCs

A 2-hour e-learning course will be the common core of the training. It comprises 7 modules: (1) training objectives; (2) review of national and international literature on SDM; (3) understanding SDM; (4) cystic fibrosis–related diabetes; (5) developing an information and decision support tool; (6) communicate benefits and risks to the patient; and (7) encourage active patient participation by means of a video of 2 simulated consultations with a doctor from the CFRC in Lyon and a patient who volunteered to take part in the project since its inception: one consultation leading to a shared decision to start insulin now and the other to start later. The e-learning will be supplemented by a presentation by one of the researchers specialized in SDM at the 3 CFRCs to answer questions/comments from the teams.

##### Component B: Individual Coaching for Physicians and Medical Team After e-Learning

This will be performed by the SDM methodologist to improve physicians’ adoption of SDM practice. In line with research on this method [33], coaching will be provided verbally. The SDM methodologist will observe 1 or 2 consultations in each of the CFRCs, providing both oral and written feedback. Her feedback will be standardized across the 3 CFRCs on the 3 essential components of SDM (bilateral information/deliberation/common agreement on the decision taken).

##### Component C: Implementing SDM in 6 Steps

The 6 steps are as follows.

1. Initial consultation with the patient to discuss diabetes treatment options, detailing the benefits and risks of each option. The physician and patient inform each other, discuss throughout the consultation with the help of the information and decision support tool, elicit their preferences, and work toward reaching an agreement on the decision to take.
2. Patient activation strategy: this patient-mediated method is based on the “Ask 3 questions” tool used in international SDM implementation studies [20]. It consists of short questions that patients can ask their doctor to enable them to participate more fully in decision-making if they so wish (ask 3 questions: what are my options? what are the possible benefits and harms of those options? how likely are the benefits and harms of each option to occur?) [20]. This tool is displayed in the waiting room before the consultation.
3. The physician gives the patient a paper version of the information and decision support tool [21].
4. The physician presents the exchanges with the patient to the CFRC team trained in SDM to obtain their feedbacks.
5. A cooling-off period of 8-15 days is required before the second consultation. During this period, the patient can discuss with his or her family, general practitioner, or any other health care professional outside the CFRC as well as with the CFRC team, and can call, based on the information and decision support tool, if he or she so wishes.
6. A second consultation takes place after this period of reflection to make the decision (in this case, to take insulin now or later). Several attitudes are possible: clear patient preference for one option, patient decides alone, refusal by the patient to choose an option, or the physician decides on the basis of the therapeutic thesaurus. It should be noted, however, that this is not the paternalistic model, as far as it is the patient who, after being informed, asks the doctor to decide; thereafter, they come to a common agreement on the decision taken (decision taken together), resulting in SDM.

##### Component D: Link With Institutional Initiatives to Promote Patient Involvement

This will be performed by means of a short questionnaire describing the current situation on the topic. At the start of the study, this questionnaire will be supplemented by a phone interview with the hospital quality manager or referent. As CFRC teams are integrated into university hospitals that implement patient engagement initiatives, it will be important to collaborate with their management teams to work together on integrating the SDM approach into these hospitals.

##### Component E: Integrating SDM Into CFRC Multidisciplinary Concertation Meetings

In order to best integrate SDM into the 3 CFRCs in the intervention group, we will organize in 2025 and in 2026 a meeting in each of the CFRCs to see how to integrate SDM into the recommendations for practice in multidisciplinary concertation meetings, and we will consider the organizational and practice specificities of each of these 3 CFRCs.

#### Standard Care

**Figure 1:**
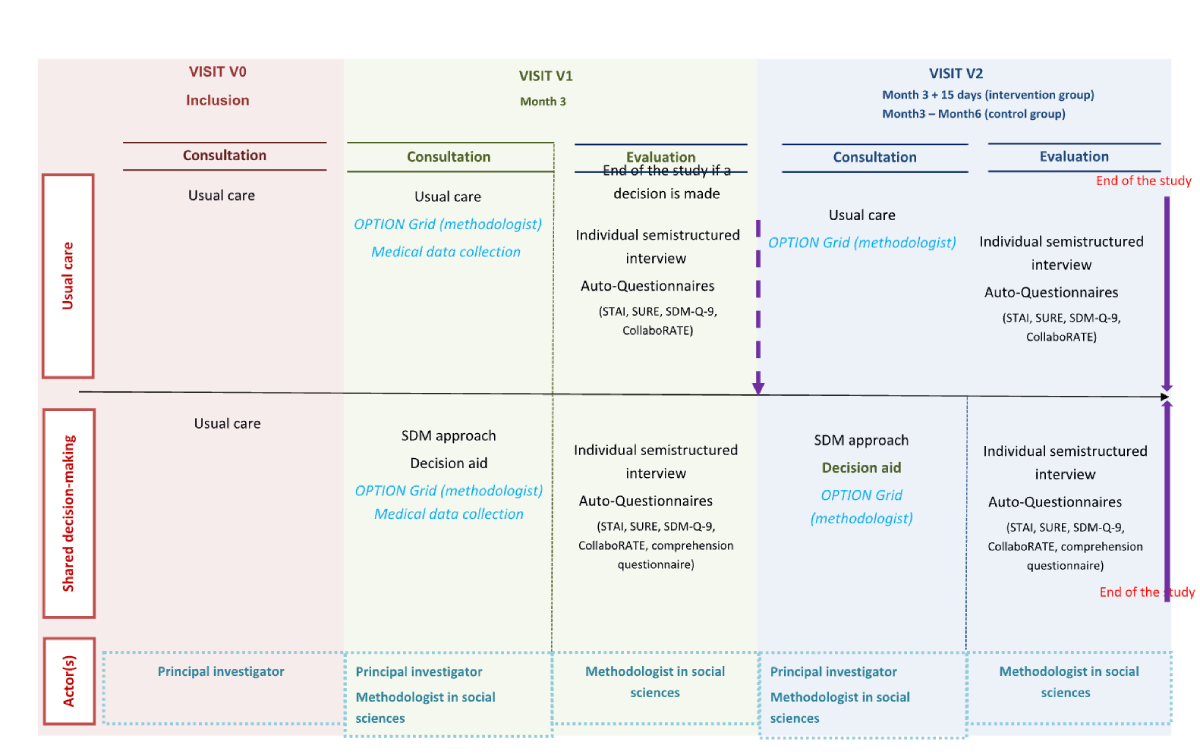
FORAIDMUCO study protocol. SDM: shared decision-making; SDM-Q-9: 9-item Shared Decision-Making Questionnaire; STAI: State-Trait Anxiety Inventory; SURE: Sure of myself, Understand information, Risk-benefit ratio, Encouragement; OPTION: Observing Patient Involvement in Shared Decision Making.

Insulin therapy decisions are made according to the usual practice defined in each CFRC, with decision-making procedures specific to each center and each CFRC physician/dietitian/nutritionist/team. The decision-making process generally involves 2 consultations (Figure 1).

### Outcomes and Measures

The outcomes are measured using the following measures.

1. Adoption or nonadoption of SDM: SDM-Q-9 [34,35], CollaboRATE questionnaire [36], OPTION (Observing Patient Involvement in Shared Decision Making) questionnaire [37,38]
2. Level of patient knowledge: knowledge questionnaire developed by the authors on the basis of their previous work [39] and the literature since no validated knowledge questionnaire exists
3. Patient anxiety level: Spielberger State-Trait Anxiety Inventory [40]
4. Patients’ level of decisional conflict: SURE questionnaire [41]
5. Patients’ experience of information and decision-making procedures and more specifically of SDM implementation
6. Individual and organizational factors influencing SDM implementation

### Inclusion Visit (V0)

When the patient comes to the CFRC following orally induced hyperglycemia, the physician will check the patient’s eligibility criteria. During this visit, the physician will present the study and give the patient the information note. The physician will record the patient’s nonopposition in the consultation report. The methodologist in social sciences must be informed of the patient’s inclusion by sending an email containing the patient’s first and last initials and the date of the V1 visit so that she can contact the investigating center to schedule her visit.

### Visit V1

In the CFRCs of the control group, the physician will present the patient with information on diabetes management according to his or her usual practice. In the CFRCs in the intervention group, the physician will present the patient with information on diabetes management according to SDM (options, benefits, and risks) by using the information and decision support tool. The patient will be “activated” prior to the consultation, inviting him/her to ask questions using the “Ask 3 questions” tool. During the V1 visit for the control and intervention groups, the investigator will fill in the patient’s clinical data and SDM-Q-9 adapted to the physician immediately after the consultation. The patient fills in the questionnaires immediately after the consultation (the questionnaires are handed over by the methodologist or a member of the CFRC). The methodologist in social sciences will observe the physician-patient interaction during the consultation and complete the OPTION questionnaire; conduct an individual semistructured interview with the patient immediately after the consultation; ensure that the patient completes the SURE, CollaboRATE, SDM-Q-9, and Spielberger State-Trait Anxiety Inventory questionnaires (if the patient does not read French, the methodologist will read the questionnaires to the patient and fill them in with his/her agreement); and collect the patient’s sociodemographic characteristics.

### Decision-Making Visit (V2)

In the CFRC control group, insulin therapy decision-making will be performed according to the physician’s usual practice. In the CFRCs of the intervention group, the decision-making visit will take place after a cooling-off period of 8 to 15 days following the V1 consultation. A discussion based on feedbacks from this period will take place between the physician and the patient, and either there is a common agreement on the decision taken (SDM) or the decision is taken by the patient or the decision is taken by the physician at the patient’s demand. During this visit, the investigator will complete the SDM-Q-9 adapted to the physician immediately after the consultation. The patient fills in the questionnaires immediately after the consultation (the questionnaires are handed over by the methodologist in social sciences or a member of CFRC). The methodologist in social sciences will observe the physician-patient interaction during the consultation and complete the OPTION questionnaire, conduct an individual semistructured interview with the patient immediately after the consultation, and ensure that the patient completes SURE, CollaboRATE, SDM-Q-9, and the Spielberger State-Trait Anxiety Inventory. If the patient does not read French, the methodologist will read the questionnaires to the patient and fill them in with his/her agreement.

In the control and intervention groups, the methodologist in social sciences will attend the face-to-face consultation (possibly by videoconference if conditions require and allow) and will conduct the individual interview with the patient immediately after the consultation. If the patient is not available, the interview can take place up to V1+48 hours. The end of the research for patients in both groups is defined by the end of the individual interview at V2 or V1 for the control group if a decision is made following the consultation.

### Health Care Professional Focus Groups

Two focus groups will be conducted in the intervention CFRCs (one at the start of the study and one at the end) and one in each of the 2 control centers by the methodologist in social sciences on the basis of a moderation guide containing the key items on which participants will be invited to discuss (barriers, facilitators of information and decision-making, dedicated time with and without SDM, information and decision support tool, etc). The discussion group will be made up of 4-8 health care professionals (physicians, nurses, physiotherapists, dietitians, psychologists, etc) depending on the center and will be as representative as possible of the CFRC’s health care professional categories. In the intervention centers, the participating health care professionals will not necessarily have taken the e-learning training course. However, the group should be identical for both discussion groups unless a member is absent or leaves.

### Data Collection and Analysis

#### Sample Size

As this is a pilot study, 40 patients will be included and interviewed over a 12-month period. The number of patients included corresponds to the active patient file of the 5 CFRCs over the 12-month inclusion period. Five physicians (1 in each center) will be involved in this study.

#### Analysis

All patients and health care professionals included in this study are in accordance with the inclusion and noninclusion criteria. The primary end point is the adoption or nonadoption of SDM from the patient’s and health care professional’s point of view. The secondary end points are patient knowledge; patient anxiety levels; patients’ level of decisional conflict; patients’ experience of information and decision-making procedures, and more specifically of SDM implementation; effect of SDM on the physician; and individual and organizational factors influencing SDM implementation.

### Statistical Methods

#### Descriptive Analysis

A descriptive analysis of the characteristics of the patient population included (age, sex, history and severity of disease) and of the health care professionals on the CFRC teams (physicians, nurses, dietitians, psychologists, physiotherapists, etc, age, sex, profession, previous training in SDM) will be performed in the 2 study groups. Quantitative characteristics will be described by mean and standard deviation or by quartiles and minimum and maximum values depending on the shape of the distribution. Qualitative characteristics will be described by the numbers and percentages in each category. The comparability of the characteristics will be checked using the chi-square test for qualitative variables and the Wilcoxon test for quantitative variables. Data on the implementation of SDM in the intervention group will also be the subject of a descriptive analysis on the proportion of patients with at least one SDM consultation and the proportion of patients with 2 SDM consultations.

#### Analysis: Primary End Point

Adoption of SDM as perceived by the patient and measured using SDM-Q-9 will be analyzed in patients with no missing data on this criterion, although an approach to managing missing data may be considered. The frequency distribution of the 6 modalities of SDM-Q-9 will be described for each group in the patient unit. A total score will be obtained by summing the scores for each of the 9 questions. A transformation will be applied to obtain a total score between 0 and 100, with 0 indicating nonadoption of SDM as perceived by the patient and conversely 100 indicating adoption of SDM as perceived by the patient. The total score will be described in each group by mean, standard deviation, median, quartiles, and range, and will be compared between the 2 groups with a nonparametric Wilcoxon test. For patients in the intervention group, the variation in the responses to each of the SDM-Q-9 questions between the 2 consultations will be tested using the McNemar test (test adapted to paired data). Total SDM-Q-9 scores between the 2 consultations will be compared using the Wilcoxon signed-rank test. The same analysis will be performed on the health care professional unit to compare the adoption of SDM as perceived by the health care professional. Discordances between patient and health care professional responses to each of the 9 questions on SDM-Q-9 will be described and tested using the McNemar test. The total SDM-Q-9 score obtained on the patient unit and the professional unit will be compared using the Wilcoxon signed-rank test.

#### Analysis: Secondary End Points

In accordance with the scoring rules of the OPTION grid, an OPTION score will be calculated for each patient-health care professional dyad if 100% of the 5 OPTION grid items have been completed. A total score between 0 and 20 will then be obtained by summing the answers to the 5 OPTION grid items between 0 and 4. The total score will be converted between 0 and 100, with high values indicating exemplary behavior by the dyad in adopting SDM. The OPTION score will be expressed as the mean and standard deviation for each group and will be compared using the Wilcoxon test.

In accordance with the scoring rules of the SURE questionnaire, a SURE score will be calculated for each patient if 100% of the 4 questionnaire items have been completed. A total score between 0 and 4 (a high value indicates a decisional conflict) will then be obtained by summing the binary responses of the 4 questionnaire items. The SURE score will be expressed as the mean and standard deviation for each group and will be compared using the Wilcoxon test. The percentage of patients whose SURE score is less than or equal to 3 (indicating a decisional conflict) will also be calculated in each group and compared using the chi-square test.

In accordance with the scoring rules of the CollaboRATE questionnaire, a CollaboRATE score will be calculated for each patient if 100% of the 3 questionnaire items have been completed. A total score between 0 and 9 (high values indicating a high level of SDM) will then be obtained by averaging the answers to the 3 questions between 0 and 9 on the questionnaire. The CollaboRATE score will be expressed as the mean and standard deviation for each group and will be compared using the Wilcoxon test.

A Spielberger State-Trait Anxiety Inventory score will be calculated for each patient if 100% of the 20 questionnaire items have been completed. A total score between 20 and 80 (high values indicating a high level of anxiety) will then be obtained by summing the scores associated with the 20 items. For questions 3, 4, 6, 7, 9, 12, 13, 14, 17, and 18, 1 point is awarded for not at all, 2 points for somewhat, 3 points for moderately, and 4 points for very much. For questions 1, 2, 5, 8, 10, 11, 15, 16, 19, and 20, the scoring will be reversed, that is, 4 points for not at all, 3 points for a little, 2 points for moderately, and 1 point for a lot. The Spielberger State-Trait Anxiety Inventory score will be expressed as the mean and standard deviation for each group and will be compared using the Wilcoxon test.

Semistructured individual interviews designed to assess the experience of information and decision-making procedures in the control and intervention groups and the effects of SDM on patients’ and health care professionals’ experience of care in the intervention groups only will be analyzed by means of a thematic content analysis conducted using NVivo 10 (QSR International) and 2 researchers participating in the study based on the interview guide developed and previously tested with patients at the CFRC in Lyon. The focus groups conducted in the control and intervention groups designed to analyze the individual and organizational factors influencing the implementation or nonimplementation of SDM will be transcribed on the basis of notes taken during the focus groups and will be analyzed on the basis of the interview guide developed.

## Results

Forty patients will be included (8 patients in each center), that is, 60 consultation observations (2 consultations per patient in the intervention groups given the modalities of the SDM process) will be conducted in 2025. Eight focus groups will be conducted in the 5 centers (2 groups in each intervention CFRC and 1 group in each control CFRC) in 2025. This qualitative corpus plus responses to the patient and physician questionnaires will make it possible to know whether the practice of SDM in CFRCs is increased by an implementation strategy and to analyze the experience of patients and their relatives regarding decision-making modalities. Analysis of the outcomes and experience of the implementation of SDM are of importance to identify the facilitators and barriers to SDM from patients’ and CFRCs’ point of view.

## Discussion

This is the first study protocol on cystic fibrosis treatment in France, which is designed to train health care professionals on SDM and implement and evaluate the outcomes of the SDM approach. Training materials and health care professionals trained in SDM could boost SDM implementation in cystic fibrosis therapy in French-speaking countries, where the so-called clinical champions in SDM are needed. Moreover, patients experiencing SDM could support and participate in the SDM acculturation process.

The aim of our study is also to propose an original study based on mixed methods combining quantitative and qualitative analyses by using validated evaluation tools (particularly in French) and to conduct the study with patients and health care professionals for whom the potential benefits are multiple: to support in decision-making concerning diabetes treatment; to improve communication between physicians, health care team, and patients; to improve the quality of experience of the decision-making steps; and to acculturate health care professionals to SDM and formalize their practice, thanks to SDM training.

The protocol and information and the decision support tool developed could serve as a basis for other situations in the field of cystic fibrosis, as in the case of lung transplantation, subject to the adaptations to be made. There are many opportunities for SDM in cystic fibrosis, but little is known about patients’ experience of SDM [42]. The training materials and the teams already trained could prove to be the key to future success, with those trained becoming trainers for other centers. International comparisons could also be developed, notably within the framework of the La Collaboration Francophone sur la Prise de Décision Partagée FREeDOM (French collaboration on SDM), which brings together health care professionals, patients, researchers, and public decision makers, notably from Quebec, Switzerland, Belgium, and France, interested in adapting the approach developed to their countries/context.

Although this is the first French study to train health care professionals and implement and evaluate SDM in cystic fibrosis treatment, compared to the usual clinical practice, the number of CFRCs involved is limited, which will potentially limit the generalizability of the results obtained. This study will nonetheless be conducted at the national level. In addition, only a small number of patients with cystic fibrosis are concerned about the development of diabetes, which could impact the duration of our project in order to obtain the desired number of patients.

## Appendix

Multimedia Appendix 1 StaRi (Standards for Reporting Implementation Studies) checklist.

## Abbreviations

CFRC: cystic fibrosis reference center
FREeDOM: French collaboration on shared decision-making
MAGIC: Making Good Decisions in Collaboration
OPTION: Observing Patient Involvement in Shared Decision Making
SDM: shared decision-making
SDM-Q-9: 9-item Shared Decision-Making Questionnaire
StaRi: Standards for Reporting Implementation Studies
SURE: Sure of myself, Understand information, Risk-benefit ratio, Encouragement

## Data Availability

Datasets that will be generated and analyzed during this study will be available from the corresponding author on reasonable request.
